# Optimal timing of antibiotics administration for sepsis or septic shock in the emergency department

**DOI:** 10.1186/s12873-026-01471-5

**Published:** 2026-01-19

**Authors:** Ming-Shun Hsieh, Kuan-Chih Chiu, Shu-Hui Liao, Vivian Chia-Rong Hsieh, Sung-Yuan Hu, Chorng-Kuang How

**Affiliations:** 1 Department of Emergency Medicine, Taoyuan Branch, Taipei Veterans General Hospital, Taoyuan, Taiwan; 2 Department of Emergency Medicine, Taipei Veterans General Hospital, Taipei, Taiwan; 3 School of Medicine, National Yang Ming Chiao Tung University, Taipei, Taiwan; 4 Department of Emergency Medicine, Taichung Veterans General Hospital, Taichung, Taiwan; 5 College of Public Health, National Taiwan University, Taipei, Taiwan; 6 Department of Pathology and Laboratory, Taoyuan Branch, Taipei Veterans General Hospital, Taoyuan, Taiwan; 7 Hsin Sheng Junior College of Medical Care and Management, Taoyuan, Taiwan; 8 Department of Health Services Administration, China Medical University, Taichung, Taiwan; 9 Department of Post-Baccalaureate Medicine, College of Medicine, National Chung Hsing University, Taichung, Taiwan; 10 School of Medicine, Chung Shan Medical University, Taichung, Taiwan

**Keywords:** Sepsis, Septic shock, Time to antibiotics (T2A)

## Abstract

**Objectives:**

Early antibiotic administration is considered an important component of sepsis management, yet the optimal time-to-antibiotics (T2A) remains uncertain. This study examined the association between T2A and in-hospital mortality among patients with sepsis and septic shock and explored how this relationship varies across different time intervals.

**Methods:**

We conducted a retrospective cohort analysis of emergency department patients with sepsis from 1998 to 2022. Patients were dichotomized into two groups (T2A ≤ 1 h vs. T2A > 1 h), and clinical characteristics and mortality outcomes were compared. Cox proportional hazards models were used to assess the association between T2A and in-hospital mortality, adjusting for illness severity and other covariates. A non-linear Cox regression model was further applied to characterize the time-dependent relationship between T2A and mortality risk.

**Results:**

A total of 15,317 patients were included. In-hospital mortality was 35.9% for patients receiving antibiotics within 1 h and 47.4% for those treated after 1 h (*P* < 0.001). After adjustment, T2A ≤ 1 h remained associated with lower mortality (adjusted HR = 0.936; 95% CI, 0.891–0.982). Non-linear modeling suggested that mortality risk was generally lower when antibiotics were administered within approximately 3 h, with the lowest estimated hazard observed at around 0.5 h; risk increased more noticeably beyond the 3-hour mark. These patterns were consistent across patients with and without septic shock.

**Conclusion:**

In this large retrospective cohort, shorter time-to-antibiotics was *associated* with lower in-hospital mortality, with the most favorable estimates occurring within the first hour and a gradual attenuation of benefit approaching 3 h. These findings provide insight into the time-dependent relationship between antibiotic administration and outcomes in sepsis that warrants further validation before being incorporated into clinical practice recommendations.

**Clinical trial number:**

Not applicable.

**Supplementary Information:**

The online version contains supplementary material available at 10.1186/s12873-026-01471-5.

## Background

Sepsis is an acute, heterogeneous condition that affects 1.7 million adults in the United States annually and contributes to more than 250,000 deaths, with post-survival complications in up to 70% of patients [1]. Characterized by excessive inflammation, metabolic disturbance, and immunosuppression, sepsis is defined as a life-threatening organ dysfunction caused by dysregulated host response to infection [2, 3] while septic shock is a more advanced complication of sepsis that potentially leads to dangerously low blood pressure and multiple organ failure. Treatment is often empiric and involves fluid resuscitation, broad-spectrum antibiotics, source control of infection, and vasopressor support [4].

One of the most important strategies to reduce in-hospital mortality in patients with sepsis or septic shock is the early administration of antibiotics [5]. However, the optimal time to antibiotics (T2A) administration remains unclear because of weak supporting evidence, even in the latest 2021 Surviving Sepsis Campaign (SSC) guideline [6, 7]. Following the 2018 SSC revisions, the 3-hour and 6-hour bundles were combined into a single 1-hour bundle. Ideally, the goal is to begin both resuscitation and management immediately, as progressive delays may lead to higher mortality [8]. Introducing the 1-hour sepsis bundle is currently highly recommended due to its significant reduction in in-hospital mortality. Multiple studies have shown that adhering to SSC bundle treatment is linked to decreased mortality in sepsis [9, 10].

The Infectious Diseases Society of America agreed that aggressive management with early administration of empirical antibiotics (within 1 h) may be helpful for patients with septic shock, but not for sepsis patients without shock [11]. The rationale for this position was that septic shock is a much severe form of sepsis and that unnecessary exposure to broad-spectrum antibiotics in sepsis without shock is potentially harmful (e.g., in the case of an allergic reaction). The Task Force on Early Care of Adult Suspected Sepsis in the Emergency Department (ED) and Out-of-Hospital Environment, recommended prompt administration of antibiotics in the ED, but a very short time threshold was reserved for those with infection and shock. The Task Force also acknowledged that there were still insufficient data to support a specific time threshold. However, it essentially supported an early T2A administration once sepsis was diagnosed or deemed highly possible, especially in patients with suspected septic shock [12]. The American College of Emergency Physicians (ACEP) also mentioned a lack of evidence to recommend a strict T2A administration in patients with sepsis [13]. Furthermore, the coordination of administering antibiotics in a timely manner necessitated significant exertion and allocation of resources. Additionally, this practice may result in inequitable resource distribution among patients who did not have sepsis [14].

This study compared the in-hospital mortality in patients with sepsis with different T2A administration. Most importantly, we aimed to identify the T2A that would equally benefit sepsis patients with or without shock. In our experience, the clinical situation of sepsis patients is highly variable, and the condition of these patients can deteriorate into septic shock at any time even if they are receiving treatment. Therefore, we aimed to establish an optimal T2A timeframe which should be taken into consideration within the latest sepsis treatment policies.

## Methods

### Study design and data source

This retrospective cohort study analyzed emergency department (ED) data from a single tertiary medical center in Taiwan between 1998 and 2022. A single-center design was selected to ensure consistency in sepsis management practices, thereby reducing inter-hospital variability and minimizing definitional and operational heterogeneity that could confound time-to-antibiotics (T2A) analyses. As sepsis definitions evolved substantially during the study period, with Surviving Sepsis Campaign (SSC) guidelines released in 2004, 2012, 2016, 2018, and 2021, we adopted a framework that preserved historical diagnostic validity while enabling modern severity adjustment [15, 16].

#### Sepsis case inclusion

This was based on the clinical standards used at the time of presentation. From 1998 to 2015, sepsis was identified using systemic inflammatory response syndrome (SIRS) criteria plus suspected infection, which reflected the prevailing Sepsis-1/Sepsis-2 definitions. Applying Sepsis-3 criteria retrospectively to this period would have excluded many patients who were appropriately diagnosed and treated according to contemporaneous practice and would have introduced substantial retrospective definition bias. Therefore, SIRS-based definitions were used for inclusion across the full study period to maintain epidemiologic coherence. To enable consistent adjustment for illness severity, SOFA scores were retrospectively reconstructed for all patients using raw physiological and laboratory data (bilirubin, creatinine, platelet count, blood pressure, and Glasgow Coma Scale). SOFA was not used as an inclusion criterion but was incorporated as a continuous covariate in multivariable models to harmonize severity assessment across the 24-year span.

#### Septic shock definition

Septic shock was classified using a harmonized approach that aligned with clinical practice across eras. Shock was identified based on documentation of hypotension requiring vasopressors and evidence of organ hypoperfusion within the first 6 h of ED presentation, consistent with operational elements of the 6-hour sepsis bundle [17]. When available in later years, lactate criteria consistent with Sepsis-3 definitions were incorporated, but Sepsis-3 criteria were not applied retroactively for case inclusion. This approach ensured consistent identification of clinically meaningful shock states despite definitional evolution.

### Inclusion and exclusion criteria

Patients were eligible if they met the following criteria, (i) Age > 18 years; (ii) Complete hospital outcome data (survival or mortality); (iii) Admission from the ED with a principal diagnosis of sepsis; (iv) No ED or hospital re-admission within 60 days; (v) Time-to-antibiotics (T2A) < 24 h; and (vi) No do-not-resuscitate (DNR) order prior to ED arrival. Re-admissions were excluded to minimize confounding related to inadequate initial treatment or post-discharge complications. Additional exclusions included transfers from other hospitals/EDs and cases receiving inappropriate antibiotic therapy (IAAT). IAAT was defined as non-concordance with the hospital’s annually updated antibiotic guidelines, which are based on the institution-specific antibiogram and developed by an infectious disease–led Infection Control Committee. Appropriateness was assessed using a decision-based stewardship framework, evaluating guideline concordance at the time of prescription rather than relying solely on retrospective culture results. All initial antibiotic regimens were reviewed by two emergency physicians and one intensivist using a standardized protocol, with independent validation by an infectious disease specialist confirming that clear guideline violations were uncommon and not significantly associated with in-hospital mortality.

### Study variables

The following demographic and clinical information was collected for all study participants: age, sex, baseline comorbidities, Charlson comorbidity index (CCI) score, SOFA score, diagnosis of septic shock, in-hospital mortality, length of ED stay, intensive care unit (ICU) admission, diagnosis of acute respiratory failure, the main infection sites, and laboratory data (white blood cell (WBC) (/µL), platelet (×103/µL), albumin (g/dL), total bilirubin (mg/dL), creatinine (mg/dL), c-reactive protein (CRP) (mg/dL), procalcitonin (ng/mL), lactate (mmol/L).

### Definition of T2A and data retrieval

Time-to-Antibiotics (T2A) was defined as the interval between ED triage registration time and the time of first intravenous antibiotic administration as documented in the nursing Medication Administration Record (MAR). All timestamps were extracted from the electronic clinical database of Taichung Veterans General Hospital under Institutional Review Board approval, and T2A values were calculated and categorized for analysis.

### T2A grouping and study endpoints

For the primary comparison, patients were divided into two groups: T2A ≤ 1 h and T2A > 1 h. To further examine dose–response patterns, the delayed-treatment group (T2A > 1 h) was stratified into finer categories: 1–2 h, 2–3 h, and > 3 h. In-hospital mortality (at 7, 14, and 28 days) served as the primary outcomes for evaluating whether progressively earlier antibiotic administration improved survival in sepsis.

### Statistical analysis

Missing data were addressed initially to ensure a complete analytical dataset. Subsequently, we conducted preliminary group-wise comparisons for all variables, followed by the relevant association analyses. The specific methodological procedures are detailed in the subsections that follow.

#### Handling of missing data

As several biomarkers (e.g., lactate, procalcitonin) were not routinely measured in earlier years, missingness followed missing at random (MAR) / missing not at random (MNAR) patterns rather than being completely random. To avoid the substantial selection bias that would result from complete-case analysis, we applied k-nearest neighbors (KNN) imputation, k = 5, using normalized Euclidean distance and variables with > 98% completeness (age, WBC, bilirubin, MAP, creatinine, CCI) [17, 18]. This non-parametric approach preserves physiological correlations and is robust to outliers, allowing us to retain the full cohort of 15,317 patients for analysis.

#### Covariate analysis

For categorical variables chi-square test or Fisher’s exact test were used, when appropriate, while for continuous variables T-test was used. In the descriptive analysis, values were presented as numbers, and percentages for categorical variables, and means (with standard deviation (SD)) for continuous variables. P-values < 0.05 were considered statistically significant.

#### Univariate and multivariate analysis by Cox regression

Univariate and multivariate Cox regression analyses were performed to determine the crude and adjusted hazard ratio (HR) with 95% confidence interval (CI) for in-hospital mortality in both stages. Results were adjusted for demographic characteristics, CCI score, SOFA score, septic shock, acute respiratory failure, acute kidney injury, ICU admission, main infection site, and laboratory data. In addition to relative measures, crude and adjusted absolute risk differences (ARDs) were calculated to quantify the absolute mortality differences associated with T2A categories. Kaplan–Meier analysis with the log-rank test was also performed to describe the risk (hazard) of in-hospital mortality between the groups in stage 1 (T2A ≤ 1 h vs. > 1 h) and stage 2 (T2A ≤ 1 h, between 1 and 2 h, between 2 and 3 h, and > 3 h). All statistical data were analyzed using R 4.1.0 software.

#### Non-linear modeling

We used a non-linear Cox proportional hazards model to evaluate the association between T2A and in-hospital mortality, acknowledging that the effect of antibiotic delay is unlikely to follow a strictly linear pattern [19–21]. To determine the optimal functional form, we compared Akaike Information Criterion (AIC) values across natural cubic splines, B-splines, penalized splines, and polynomial functions with two to five degrees of freedom; a natural cubic spline with two degrees of freedom provided the best fit and was therefore selected. While T2A was modeled non-linearly, all potential confounders, including age, sex, Charlson Comorbidity Index, SOFA score, respiratory failure, acute kidney injury, ED length of stay, and ICU admission, were incorporated as linear terms. This approach preserves model stability and statistical power while allowing flexible estimation of the time-dependent risk associated with delayed antibiotic administration.

### Ethics approval and informed consent

The study was conducted in accordance with the STROBE reporting guidelines for observational studies [22]. This study was approved by the institutional review board of Taichung Veterans General Hospital (IRB No. CE22240B). Due to the anonymization of patient identification to safeguard privacy prior to data dissemination, the study was deemed exempt from obtaining informed consent from participants. Meanwhile, because of the observational and retrospective design, the clinical trial registration was not applicable in this study. Human Ethics and Consent to Participate declarations were not applicable.

## Results

**Fig. 1:**
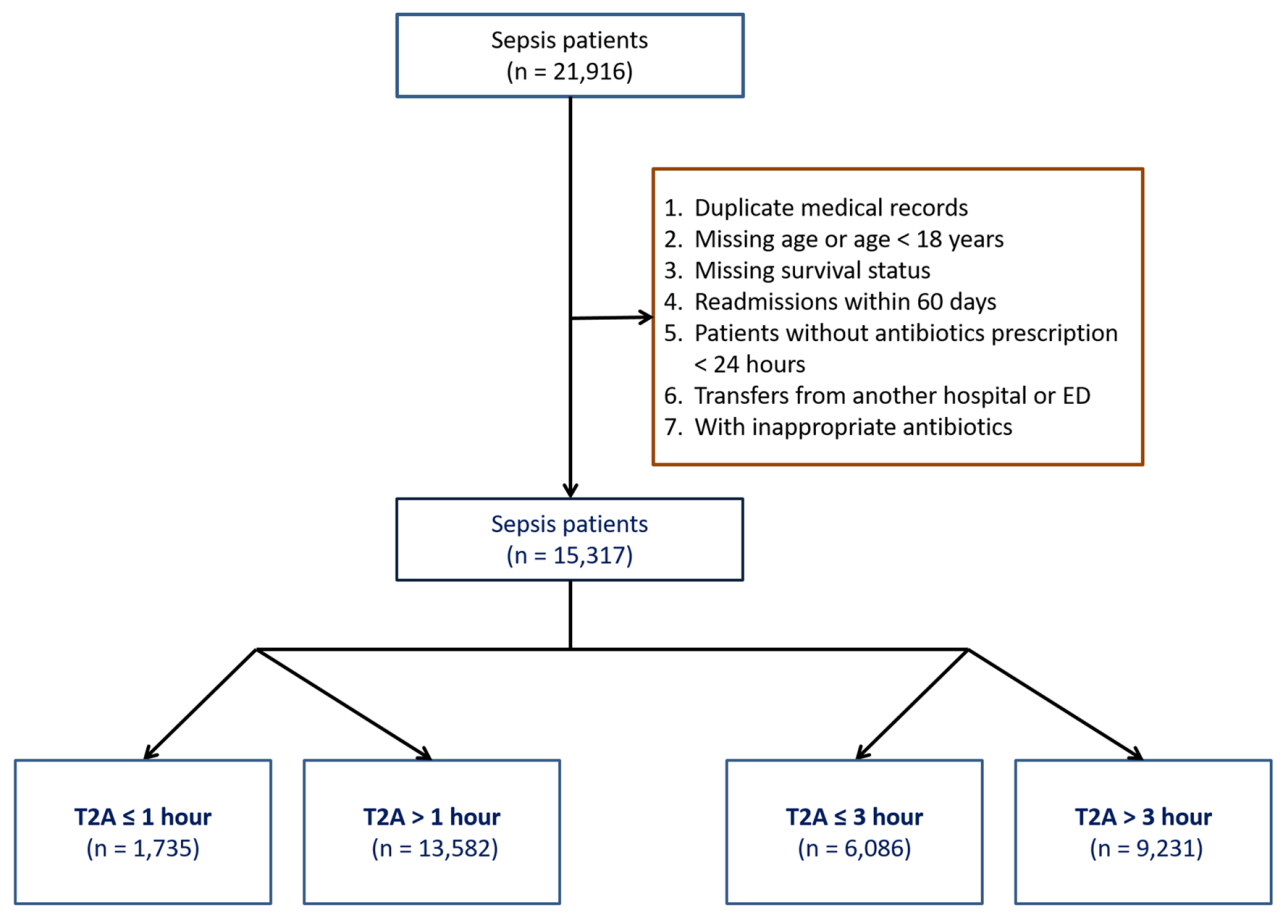
Selection algorithm to enroll sepsis patients from the emergency department sepsis cohort between 1998 and 2022. Patients were screened based on age, availability of outcome data, time-to-antibiotics (T2A) < 24 h, absence of pre-existing do-not-resuscitate orders, and non-transfer status. Cases receiving inappropriate antibiotic therapy (IAAT), as determined by institutional guidelines, were excluded. The final analytic sample included 15,317 patients. Brown box: exclusion criteria

Overall, 15,317 patients with sepsis admitted to the ED were identified (Fig. 1). The T2A ≤ 1 h and > 1 h groups contained 1,735 (11.3%) and 13,582 (88.7%) patients, respectively (Table 1). The overall mean age was 67.5 years, and 62.7% were male. The in-hospital mortality rate was 35.9% in the T2A ≤ 1 h group and 47.4% in the T2A > 1 h group. (For comparison, the annual in-hospital mortality among sepsis patients from 2009 to 2022 is shown in Figure S1; it ranged from 37.0% to 49.4%.) The mean CCI score was 4.45 ± 3.36 and 4.75 ± 3.28 in the T2A ≤ 1 h and > 1 h groups, respectively (*P* < 0.001), and the mean SOFA score was 9.40 ± 3.39 and 9.75 ± 3.29 (*P* < 0.001). Furthermore, 791 of the 1,735 (45.6%) patients in the T2A ≤ 1 h group had septic shock; however, 7,621 of the 13,582 (56.1%) patients in the T2A > 1 h group had septic shock (*P* < 0.001). Six hundred and ninety-seven patients (40.2%) required ICU admission in the T2A ≤ 1 h group compared with 6,415 patients (47.2%) in the T2A > 1 h group. The T2A ≤ 1 h group presented with worse laboratory values, particularly creatinine, C-reactive protein, and procalcitonin (all *P* < 0.05) (Table 1).

**Table 1:** Comparison of baseline demographic and clinical characteristics between early and delayed antibiotic administration groups

Variables	T2A < = 1 h (*n* = 1,735)	T2A > 1 h (*n* = 13,582)	*p* ^1^
Demographic characteristics			
Age [median (IQR)]	69.41 (24.98)	70.72 (23.36)	**0.02**
Sex [Males, n (%)]	1121 (64.61)	8486 (62.48)	0.09
BMI [median (IQR)]	23.39 (5.31)	23.05 (5.54)	**0.004**
**Mortality [n (%)]**	623 (35.91)	6438 (47.4)	**< 0.001**
**Comorbidities [n (%)]**			
Myocardial infarction	122 (7.03)	1015 (7.47)	0.54
Congestive heart failure (CHF)	267 (15.39)	2110 (15.54)	0.9
Peripheral vascular disease	121 (6.97)	760 (5.6)	**0.02**
Cerebrovascular disease	402 (23.17)	3427 (25.23)	0.07
Dementia	163 (9.39)	1305 (9.61)	0.81
Chronic pulmonary disease (COPD)	466 (26.86)	3653 (26.9)	1
Rheumatic disease	109 (6.28)	812 (5.98)	0.65
Peptic ulcer disease	512 (29.51)	4155 (30.59)	0.37
Mild liver disease	190 (10.95)	1433 (10.55)	0.64
Diabetes without chronic complication	368 (21.21)	3043 (22.4)	0.27
Diabetes with chronic complication	188 (10.84)	1526 (11.24)	0.65
Hemiplegia or paraplegia	50 (2.88)	389 (2.86)	1
Renal disease	574 (33.08)	4354 (32.06)	0.4
ESKD	88 (5.07)	592 (4.36)	0.19
CKD	486 (28.01)	3762 (27.7)	0.81
Any malignancy, including lymphoma and leukemia, except malignant neoplasm of skin	481 (27.72)	3553 (26.16)	0.17
Moderate or severe liver disease	113 (6.51)	1037 (7.64)	0.1
Metastatic solid tumor	340 (19.6)	3296 (24.27)	**< 0.001**
AIDS/HIV	5 (0.29)	44 (0.32)	0.98
Charlson comorbidity score [CCI, median (IQR)]	4 (5)	5 (5)	**< 0.001**
**SOFA score [median (IQR)]**	9 (5)	10 (5)	**< 0.001**
**Septic shock (Inotropic or vasopressor agent use) [n (%)]**	791 (45.59)	7621 (56.11)	**< 0.001**
Dopamine	370 (21.33)	4568 (33.63)	**< 0.001**
Dobutamine	14 (0.81)	257 (1.89)	**0.002**
Norepinephrine	650 (37.46)	5862 (43.16)	**< 0.001**
**Respiratory failure [n (%)]**	572 (32.97)	5203 (38.31)	**< 0.001**
**Acute kidney injury (AKI) [n (%)]**	148 (9.01)	1496 (11.43)	**0.004**
**Length of ED stay [hours**,** median (IQR)]**	20.01 (29.42)	17.27 (30.31)	**< 0.001**
**ICU admission [n (%)]**	697 (40.17)	6415 (47.23)	**< 0.001**
**Procedures [n (%)]**			
Endotracheal intubation	258 (14.87)	2896 (21.32)	**< 0.001**
Non-inavasive positive ventilation	426 (24.55)	3530 (25.99)	0.21
Tracheostomy	103 (5.94)	791 (5.82)	0.89
Upper endoscopy	253 (14.58)	2196 (16.17)	0.1
Lower endoscopy	56 (3.23)	409 (3.01)	0.67
Cardiac catheterization	63 (3.63)	511 (3.76)	0.84
Brain computed tomography	506 (29.16)	4582 (33.74)	**< 0.001**
Brain magnetic resonance image	166 (9.57)	1310 (9.65)	0.95
Central venous catheter insertion	717 (41.33)	6653 (48.98)	**< 0.001**
Hemodialysis	230 (13.26)	1814 (13.36)	0.94
Urgent	142 (8.18)	1222 (9)	0.28
Maintenance	88 (5.07)	592 (4.36)	0.19
Blood transfusion	933 (53.78)	8500 (62.58)	**< 0.001**
**Main infection sites [n (%)]**			
Central nervous	20 (1.15)	124 (0.91)	0.4
Respiratory	605 (34.87)	5318 (39.15)	**< 0.001**
Cardiovascular	26 (1.5)	130 (0.96)	**0.05**
Gastrointestinal/biliary tract	363 (20.92)	2449 (18.03)	**0.004**
Genitourinary	547 (31.53)	3708 (27.3)	**< 0.001**
Soft tissue/musculoskeletal	83 (4.78)	463 (3.41)	**0.005**
Device-related	39 (2.25)	366 (2.69)	0.31
Others	348 (20.06)	2517 (18.53)	0.13
**Laboratory data [median (IQR)]**			
WBC (/µL)	12,230 (9915)	11,110 (9400)	**< 0.001**
Hemoglobin (g/dL)	11.5 (4.6)	11.3 (4.3)	0.07
Platelet (×10^3^/µL)	187 (146)	187 (156)	0.13
Albumin (g/dL)	2.7 (0.9)	2.8 (1)	**0.03**
Total Bilirubin (mg/dL)	0.7 (0.98)	0.7 (0.9)	0.12
Creatinine (mg/dL)	1.3 (1.48)	1.4 (1.58)	**0.03**
CRP (mg/dL)	10.12 (13.97)	9.49 (14.51)	**0.004**
Procalcitonin (ng/mL)	3.68 (18.35)	2.35 (13.62)	**0.008**
Lactate (mmol/L)	20.6 (22.45)	21.4 (23.3)	**0.01**

^1^ Mann–Whitney U test for continuous variables and *χ*^2^ test for categorical variables. *p* < 0.05 were shown in bold

Table 2 presents a more granular comparison of baseline characteristics across four T2A intervals. Increasing delays in antibiotic administration were associated with progressively higher in-hospital mortality, rising from 35.9% in the ≤ 1-hour group to 50.9% in those treated after 3 h (*p* < 0.001). Patients in the 1–2 h and 2–3 h groups tended to be older and had higher prevalence of several comorbidities, including chronic pulmonary disease, cerebrovascular disease, and diabetes, compared with the earliest-treated group. The proportion of septic shock also increased steadily across groups, from 45.6% (≤ 1 h) to 57.5% (> 3 h).

**Table 2:** Comparison of baseline demographics among patients stratified by Time-to-Antibiotics (T2A) intervals

Variables	T2A < = 1 h (*n* = 1,735)	T2A 1–2 h (*n* = 2,340)	T2A 2–3 h (*n* = 2,011)	T2A > 3 h (*n* = 9,231)	*p* ^1^
Demographic characteristics					
Age [median (IQR)]	69.41 (24.98)	72.25 (23.56)	72.59 (23.66)	70.01 (23.18)	**< 0.001**
Sex [Males, n (%)]	1121 (64.61)	1467 (62.69)	1246 (61.96)	5773 (62.54)	0.35
BMI [median (IQR)]	23.39 (5.31)	23.05 (5.62)	23.15 (5.78)	23.05 (5.48)	**0.02**
**Mortality [n (%)]**	623 (35.91)	908 (38.8)	830 (41.27)	4700 (50.92)	**< 0.001**
**Comorbidities [n (%)]**					
Myocardial infarction	122 (7.03)	165 (7.05)	186 (9.25)	664 (7.19)	**0.01**
Congestive heart failure (CHF)	267 (15.39)	378 (16.15)	379 (18.85)	1353 (14.66)	**< 0.001**
Peripheral vascular disease	121 (6.97)	168 (7.18)	150 (7.46)	442 (4.79)	**< 0.001**
Cerebrovascular disease	402 (23.17)	628 (26.84)	573 (28.49)	2226 (24.11)	**< 0.001**
Dementia	163 (9.39)	316 (13.5)	235 (11.69)	754 (8.17)	**< 0.001**
Chronic pulmonary disease (COPD)	466 (26.86)	711 (30.38)	622 (30.93)	2320 (25.13)	**< 0.001**
Rheumatic disease	109 (6.28)	113 (4.83)	114 (5.67)	585 (6.34)	**0.04**
Peptic ulcer disease	512 (29.51)	681 (29.1)	638 (31.73)	2836 (30.72)	0.2
Mild liver disease	190 (10.95)	238 (10.17)	236 (11.74)	959 (10.39)	0.28
Diabetes without chronic complication	368 (21.21)	538 (22.99)	505 (25.11)	2000 (21.67)	**0.004**
Diabetes with chronic complication	188 (10.84)	290 (12.39)	262 (13.03)	974 (10.55)	**0.003**
Hemiplegia or paraplegia	50 (2.88)	73 (3.12)	70 (3.48)	246 (2.66)	0.2
Renal disease	574 (33.08)	799 (34.15)	700 (34.81)	2855 (30.93)	**< 0.001**
ESKD	88 (5.07)	96 (4.1)	80 (3.98)	416 (4.51)	0.34
CKD	486 (28.01)	703 (30.04)	620 (30.83)	2439 (26.42)	**< 0.001**
Any malignancy, including lymphoma and leukemia, except malignant neoplasm of skin	481 (27.72)	598 (25.56)	498 (24.76)	2457 (26.62)	0.15
Moderate or severe liver disease	113 (6.51)	117 (5)	141 (7.01)	779 (8.44)	**< 0.001**
Metastatic solid tumor	340 (19.6)	483 (20.64)	407 (20.24)	2406 (26.06)	**< 0.001**
AIDS/HIV	5 (0.29)	6 (0.26)	3 (0.15)	35 (0.38)	0.36
Charlson comorbidity score [CCI, median (IQR)]	4 (5)	4 (5)	5 (5)	5 (5)	**< 0.001**
**SOFA score [median (IQR)]**	9 (5)	10 (5)	10 (5)	10 (5)	**< 0.001**
**Septic shock (Inotropic or vasopressor agent use) [n (%)]**	791 (45.59)	1251 (53.46)	1062 (52.81)	5308 (57.5)	**< 0.001**
Dopamine	370 (21.33)	523 (22.35)	487 (24.22)	3558 (38.54)	**< 0.001**
Dobutamine	14 (0.81)	34 (1.45)	33 (1.64)	190 (2.06)	**0.002**
Norepinephrine	650 (37.46)	1076 (45.98)	883 (43.91)	3903 (42.28)	**< 0.001**
**Respiratory failure [n (%)]**	572 (32.97)	853 (36.45)	699 (34.76)	3651 (39.55)	**< 0.001**
**Acute kidney injury (AKI) [n (%)]**	148 (9.01)	235 (10.47)	212 (11.06)	1049 (11.75)	**0.008**
**Length of ED stay [hours**,** median (IQR)]**	20.01 (29.42)	18.49 (25.49)	20.31 (31.95)	15.64 (31.67)	**< 0.001**
**ICU admission [n (%)]**	697 (40.17)	1195 (51.07)	932 (46.35)	4288 (46.45)	**< 0.001**
**Procedures [n (%)]**					
Endotracheal intubation	258 (14.87)	383 (16.37)	341 (16.96)	2172 (23.53)	**< 0.001**
Non-inavasive positive ventilation	426 (24.55)	654 (27.95)	499 (24.81)	2377 (25.75)	**0.04**
Tracheostomy	103 (5.94)	153 (6.54)	111 (5.52)	527 (5.71)	0.43
Upper endoscopy	253 (14.58)	381 (16.28)	320 (15.91)	1495 (16.2)	0.39
Lower endoscopy	56 (3.23)	66 (2.82)	68 (3.38)	275 (2.98)	0.68
Cardiac catheterization	63 (3.63)	93 (3.97)	87 (4.33)	331 (3.59)	0.4
Brain computed tomography	506 (29.16)	734 (31.37)	580 (28.84)	3268 (35.4)	**< 0.001**
Brain magnetic resonance image	166 (9.57)	215 (9.19)	164 (8.16)	931 (10.09)	0.05
Central venous catheter insertion	717 (41.33)	1118 (47.78)	923 (45.9)	4612 (49.96)	**< 0.001**
Hemodialysis	230 (13.26)	311 (13.29)	263 (13.08)	1240 (13.43)	0.98
Urgent	142 (8.18)	215 (9.19)	183 (9.1)	824 (8.93)	0.7
Maintenance	88 (5.07)	96 (4.1)	80 (3.98)	416 (4.51)	0.34
Blood transfusion	933 (53.78)	1360 (58.12)	1136 (56.49)	6004 (65.04)	**< 0.001**
**Main infection sites [n (%)]**					
Central nervous	20 (1.15)	27 (1.15)	9 (0.45)	88 (0.95)	0.06
Respiratory	605 (34.87)	1045 (44.66)	869 (43.21)	3404 (36.88)	**< 0.001**
Cardiovascular	26 (1.5)	23 (0.98)	18 (0.9)	89 (0.96)	0.21
Gastrointestinal/biliary tract	363 (20.92)	398 (17.01)	365 (18.15)	1686 (18.26)	**0.01**
Genitourinary	547 (31.53)	687 (29.36)	596 (29.64)	2425 (26.27)	**< 0.001**
Soft tissue/musculoskeletal	83 (4.78)	111 (4.74)	69 (3.43)	283 (3.07)	**< 0.001**
Device-related	39 (2.25)	54 (2.31)	46 (2.29)	266 (2.88)	0.16
Others	348 (20.06)	515 (22.01)	393 (19.54)	1609 (17.43)	**< 0.001**
**Laboratory data [median (IQR)]**					
WBC (/µL)	12,230 (9915)	12,110 (10100)	12,300 (10065)	10,620 (8880)	**< 0.001**
Hemoglobin (g/dL)	11.5 (4.6)	11.4 (4.2)	11.4 (4.2)	11.3 (4.5)	0.33
Platelet (×10^3^/µL)	187 (146)	183 (159)	183 (152)	188 (156)	0.06
Albumin (g/dL)	2.7 (0.9)	2.7 (0.8)	2.8 (0.9)	2.8 (0.9)	**< 0.001**
Total Bilirubin (mg/dL)	0.7 (0.98)	0.7 (0.8)	0.7 (1)	0.7 (0.9)	**0.04**
Creatinine (mg/dL)	1.3 (1.48)	1.4 (1.5)	1.4 (1.68)	1.35 (1.58)	**0.005**
CRP (mg/dL)	10.12 (13.97)	10.67 (14.71)	10.44 (14.48)	8.94 (14.46)	**< 0.001**
Procalcitonin (ng/mL)	3.68 (18.35)	3.34 (16.81)	2.72 (13.64)	2 (12.29)	**< 0.001**
Lactate (mmol/L)	20.6 (22.45)	21.8 (25)	21.9 (23.15)	21.2 (22.9)	**0.03**

^1^ Kruskal–wallis test for continuous variables and *χ*^2^ test for categorical variables. *p* < 0.05 were shown in bold

Markers of severity, such as respiratory failure, AKI, vasopressor use, and ICU admission, were increasingly common with longer T2A. Infection source patterns also differed by T2A category, with respiratory infections notably more frequent among those treated after 1 h. Laboratory findings showed modest but significant trends: CRP, creatinine, and bilirubin levels varied across T2A groups, while procalcitonin and lactate demonstrated decreasing values with longer T2A, likely reflecting early triage of clinically severe patients.

### Proportional Cox hazards regression analysis

In the univariate Cox analysis, patients receiving antibiotics within 1 h (T2A ≤ 1 h) had a significantly lower crude hazard of in-hospital mortality (HR = 0.849; 95% CI, 0.782–0.922; *P* < 0.001) (Table 3). After adjustment for confounders, early treatment remained associated with reduced mortality (adjusted HR = 0.936; 95% CI, 0.891–0.982; *P* < 0.001). Septic shock was strongly associated with increased mortality risk (crude HR = 1.665; adjusted HR = 1.479; both *P* < 0.001), whereas ICU admission was associated with a lower hazard of death (crude HR = 0.830; adjusted HR = 0.727; both *P* < 0.001) (Table 3). In terms of absolute effects, early antibiotic administration was associated with lower mortality at all evaluated time points. The crude absolute risk difference (ARD) for T2A ≤ 1 h versus > 1 h was − 11% at 7 days, − 7% at 14 days, and − 2% at 28 days **(**Table 3**)**. After adjustment for confounders, the corresponding adjusted ARDs were − 8% at 7 days, − 6% at 14 days, and − 3% at 28 days, indicating a modest but persistent absolute mortality difference over time (Table 3).

**Table 3:** Risk of in-hospital mortality in sepsis patients with time to antibiotics within and over an hour

Variables	Mortality					
	Crude HR (95% CI)	Crude *P*	Crude ARD	Adjusted HR (95% CI)	Adjusted *P* ^1^	Adjusted ARD
**Antibiotic prescription within 1 h (T2A ≤ 1 h vs. > 1 h)**	0.849 (0.782, 0.922)	**< 0.001**	-0.11 (7 days) -0.07 (14 days) -0.02 (28 days)	0.936 (0.891 0.982)	**< 0.001**	-0.08 (7 days) -0.06 (14 days) -0.03 (28 days)
**Demographic characteristics**						
Age	1.005 (1.003, 1.006)	**< 0.001**		1.006 (1.002, 1.009)	**< 0.001**	
Male (vs. female)	1.114 (1.060, 1.170)	**< 0.001**		0.976 (0.879, 1.083)	0.640	
**CCI score**	1.060 (1.053, 1.068)	**< 0.001**		1.038 (1.023, 1.053)	**< 0.001**	
**SOFA score**	1.102 (1.094, 1.111)	**< 0.001**		1.052 (1.023, 1.082)	**< 0.001**	
**Septic shock (yes vs. no)**	1.665 (1.577, 1.759)	**< 0.001**		1.479 (1.203, 1.817)	**< 0.001**	
**Acute respiratory failure (yes vs. no)**	0.935 (0.892, 0.981)	**0.006**		0.728 (0.659, 0.805)	**< 0.001**	
**Acute kidney injury (yes vs. no)**	1.506 (1.405, 1.613)	**< 0.001**		1.033 (0.897, 1.189)	0.660	
**ICU admission (yes vs. no)**	0.83 (0.791, 0.871)	**< 0.001**		0.727 (0.618, 0.857)	**< 0.001**	
**Main infection site (yes vs. no)**						
Central nervous system	0.786 (0.627, 0.986)	**0.04**		1.227 (0.830, 1.815)	0.300	
Respiratory	1.15 (1.097, 1.205)	**< 0.001**		0.977 (0.881, 1.083)	0.660	
Cardiovascular	0.871 (0.702, 1.080)	0.21		1.032 (0.675, 1.578)	0.880	
Gastrointestinal/biliary tract	0.953 (0.898, 1.010)	0.1		0.885 (0.782, 1.002)	0.050	
Genitourinary	0.520 (0.487, 0.555)	**< 0.001**		0.556 (0.477, 0.647)	**< 0.001**	
Soft tissue/musculoskeletal	0.579 (0.503, 0.666)	**< 0.001**		0.586 (0.440, 0.779)	**< 0.001**	
Device-related	0.562 (0.480, 0.659)	**< 0.001**		0.701 (0.504, 0.974)	**0.030**	
**Laboratory data**						
WBC (/µL)	1 (1, 1)	**< 0.001**		1 (1, 1)	**< 0.001**	
Hemoglobin (g/dL)	0.964 (0.956, 0.973)	**< 0.001**		0.979 (0.961, 0.998)	**0.030**	
Platelet (×10^3^/µL)	0.999 (0.999, 0.999)	**< 0.001**		0.999 (0.999, 0.999)	**< 0.001**	
Albumin (g/dL)	0.764 (0.737, 0.793)	**< 0.001**		0.819 (0.760, 0.884)	**< 0.001**	
Total bilirubin (mg/dL)	1.050 (1.045, 1.055)	**< 0.001**		1.036 (1.021, 1.051)	**< 0.001**	
Creatinine (mg/dL)	1.039 (1.029, 1.05)	**< 0.001**		0.984 (0.959, 1.010)	0.230	
CRP (mg/dL)	1.004 (1.001, 1.006)	**0.002**		0.999 (0.994, 1.003)	0.560	
Procalcitonin (ng/mL)	1.002 (1.001, 1.004)	**< 0.001**		1.002 (1.000, 1.004)	**0.020**	
Lactate (mmol/L)	1.009 (1.008, 1.01)	**< 0.001**		1.005 (1.003, 1.006)	**< 0.001**	

^1^ Adjusted for demographic characteristics, CCI, SOFA score, septic shock, acute respiratory failure, acute kidney injury, ICU admission, main infection site, and laboratory data. P-values < 0.05 are shown in bold

Abbreviations: CCI: Charlson comorbidity index. CRP: C-reactive protein. HR: Hazard ratio. ICU: Intensive Care Unit. SOFA: Sequential organ failure assessment. T2A: time to antibiotics. WBC: white blood cell count

When T2A was further stratified into four intervals (≤ 1 h, 1–2 h, 2–3 h, and > 3 h), a graded association with in-hospital mortality was observed (Table 4). Compared with the reference group (T2A > 3 h), patients treated within ≤ 1 h and 1–2 h had lower adjusted hazards of mortality (adjusted HR = 0.850; 95% CI, 0.721–1.003; *P* = 0.05 and adjusted HR = 0.816; 95% CI, 0.715–0.932; *P* = 0.003, respectively). For the 2–3 h interval, the adjusted HR was 0.886 (95% CI, 0.773–1.017; *P* = 0.08), indicating attenuation of the survival benefit as T2A approached 3 h. Consistent with these relative estimates, absolute mortality differences showed a similar graded pattern. Compared with T2A > 3 h, the adjusted absolute risk differences (ARDs) were − 10.6%, − 9.9%, and − 10.3% at 7 days for T2A ≤ 1 h, 1–2 h, and 2–3 h, respectively. At 14 days, the corresponding adjusted ARDs were − 8.4%, − 7.8%, and − 8.1%, while at 21 days they were − 3.5%, − 3.2%, and − 3.4%, respectively. Although absolute risk reductions persisted across early T2A intervals, the diminishing statistical significance of the adjusted HR in the 2–3 h group suggests that the mortality benefit becomes less pronounced as antibiotic administration approaches 3 h, supporting a “sooner is better” paradigm while indicating a practical threshold beyond which risk increases more sharply.

**Table 4:** In-hospital mortality in sepsis patients with time to antibiotics within 1 h, between 1 and 2 h, between 2 and 3 h, and over 3 h

Variables	Mortality						
	Crude HR (95% CI)	Crude *P*	Crude ARD	Adjusted HR (95% CI)		Adjusted *P* ^1^	Adjusted ARD
**Antibiotic prescription**							
> 3 h (**Ref**: *N* = 9,231, Events = 4700)	reference		-	reference			-
≤ 1 h (*N* = 1,735, Events = 623)	0.813 (0.748, 0.884)	**< 0.001**	-0.139 (7 days) -0.087 (14 days) -0.02 (21 days)	0.850 (0.721, 1.003)		**0.050**	-0.106 (7 days) -0.084 (14 days) -0.035 (21 days)
1–2 h (*N* = 2,340, Events = 908)	0.813 (0.758, 0.873)	**< 0.001**	-0.106 (7 days) -0.063 (14 days) -0.013 (21 days)	0.816 (0.715, 0.932)		**0.003**	-0.099 (7 days) -0.078 (14 days) -0.032 (21 days)
2–3 h (*N* = 2,011, Events = 830)	0.919 (0.854, 0.990)	**0.030**	-0.107 (7 days) -0.064 (14 days) -0.013 (21 days)	0.886 (0.773, 1.017)		**0.080**	-0.103 (7 days) -0.081 (14 days) -0.034 (21 days)
**Demographic characteristics**							
Age	1.005 (1.003, 1.006)	**< 0.001**			1.006 (1.003, 1.009)	**< 0.001**	
Males	1.114 (1.060, 1.170)	**< 0.001**			0.976 (0.880, 1.083)	0.650	
**CCI**	1.060 (1.053, 1.068)	**< 0.001**			1.038 (1.023, 1.053)	**< 0.001**	
**SOFA score**	1.102 (1.094, 1.111)	**< 0.001**			1.056 (1.027, 1.085)	**< 0.001**	
**Septic shock (yes vs. no)**	1.665 (1.577, 1.759)	**< 0.001**			1.450 (1.179, 1.782)	**< 0.001**	
**Acute respiratory failure (yes vs. no)**	0.935 (0.892, 0.981)	**0.006**			0.720 (0.651, 0.796)	**< 0.001**	
**Acute kidney injury (yes vs. no)**	1.506 (1.405, 1.613)	**< 0.001**			1.032 (0.896, 1.189)	0.660	
**ICU admission (yes vs. no)**	0.803 (0.791, 0.871)	**< 0.001**			0.727 (0.617, 0.856)	**< 0.001**	
**Main infection site (yes vs. no)**							
Central nervous	0.786 (0.627, 0.986)	**0.040**			1.227 (0.830, 1.814)	0.310	
Respiratory	1.150 (1.097, 1.205)	**< 0.001**			0.994 (0.896, 1.103)	0.910	
Cardiovascular	0.871 (0.702, 1.080)	0.210			1.029 (0.673, 1.572)	0.900	
Gastrointestinal/biliary tract	0.953 (0.898, 1.010)	0.100			0.890 (0.786, 1.007)	0.060	
Genitourinary	0.520 (0.487, 0.555)	**< 0.001**			0.558 (0.480, 0.650)	**< 0.001**	
Soft tissue/musculoskeletal	0.579 (0.503, 0.666)	**< 0.001**			0.595 (0.447, 0.792)	**< 0.001**	
Device-related	0.562 (0.480, 0.659)	**< 0.001**			0.697 (0.501, 0.968)	**0.030**	
**Laboratory data**							
WBC (/µL)	1 (1, 1)	**< 0.001**			1 (1, 1)	**< 0.001**	
Hemoglobin (g/dL)	0.964 (0.956, 0.973)	**< 0.001**			0.981 (0.963, 1)	**0.050**	
Platelet (×10^3^/µL)	0.999 (0.999, 0.999)	**< 0.001**			0.999 (0.999, 0.999)	**< 0.001**	
Albumin (g/dL)	0.764 (0.737, 0.793)	**< 0.001**			0.817 (0.757, 0.881)	**< 0.001**	
Total bilirubin (mg/dL)	1.050(1.045, 1.055)	**< 0.001**			1.034 (1.019, 1.049)	**< 0.001**	
Creatinine (mg/dL)	1.039 (1.029, 1.050)	**< 0.001**			0.983 (0.958, 1.008)	0.190	
CRP (mg/dL)	1.004 (1.001, 1.006)	**0.002**			0.999 (0.994, 1.004)	0.680	
Procalcitonin (ng/mL)	1.002 (1.001, 1.004)	**< 0.001**			1.002 (1, 1.004)	**0.020**	
Lactate (mmol/L)	1.009 (1.008, 1.010)	**< 0.001**			1.005 (1.003, 1.006)	**< 0.001**	

^1^ Adjusted for demographic characteristics, CCI, SOFA score, septic shock, acute respiratory failure, acute kidney injury, ICU admission, main infection site, and laboratory data. P-values < 0.05 are shown in bold

Events: in hospital mortality, Ref: Reference group, CCI score: Charlson comorbidity index score. CRP: C-reactive protein. HR: Hazard ratio. ICU: Intensive Care Unit. SOFA: Sequential organ failure assessment. WBC: white blood cell count

Overall, early antibiotic administration was associated with modest but clinically meaningful absolute reductions in mortality, particularly within the first 7–14 days, when the adjusted absolute risk differences were greatest. Given the high baseline mortality of sepsis, these absolute effects may translate into important population-level benefits, while reinforcing that antibiotic timing is one component of comprehensive sepsis care rather than a sole determinant of outcome.

### Kaplan Meier analysis

**Fig. 2:**
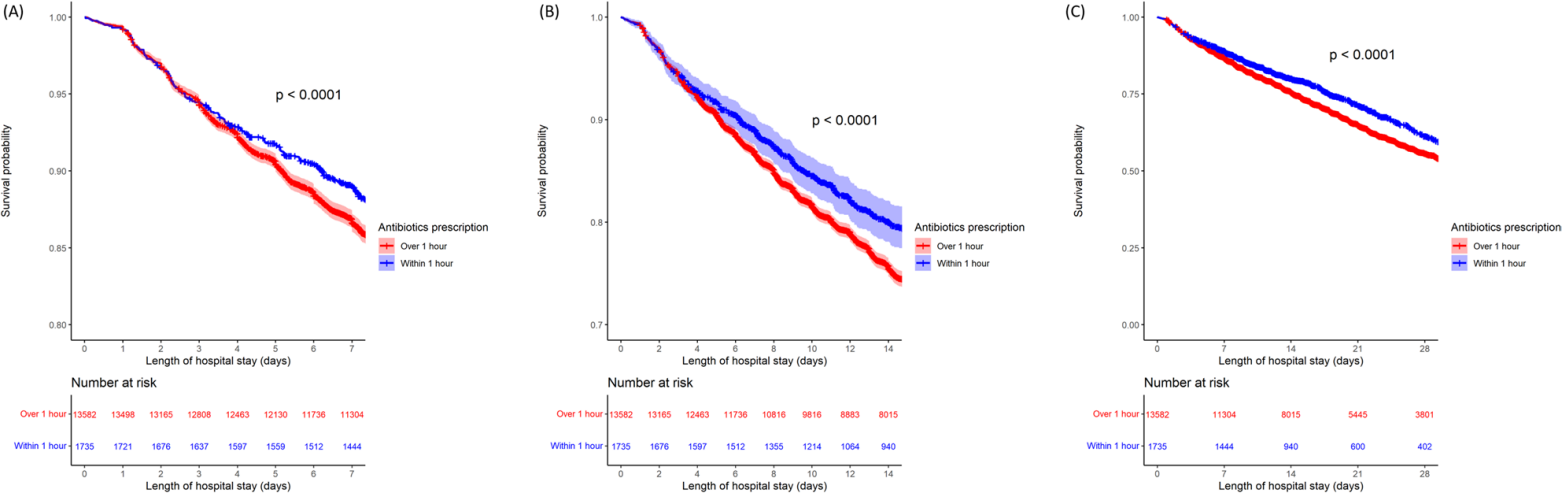
Kaplan–Meier analysis with the log-rank test. (**A**) Survival probability in sepsis patients for the first 7 days with time to antibiotics (T2A) within and over 1 h. (**B**) Survival probability in sepsis patients for the first 14 days with T2A within and over 1 h. (**C**) Survival probability in sepsis patients for the first 28 days with T2A within and over 1 h

**Fig. 3:**
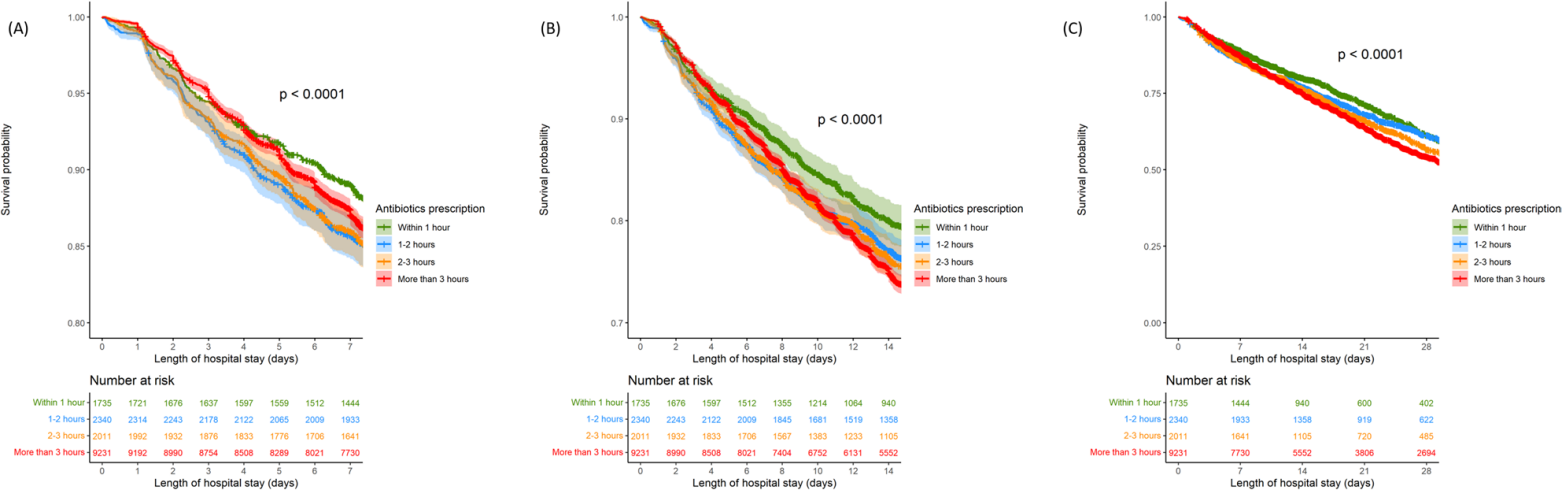
Kaplan–Meier analysis with the log-rank test with further stratification of time to antibiotics (T2A) administration. (**A**) Survival probability in sepsis patients for the first 7 days with T2A within and over 3 h. (**B**) Survival probability in sepsis patients for the first 14 days with T2A within and over 3 h. (**C**) Survival probability in sepsis patients for the first 28 days with T2A within and over 3 h

In the Kaplan–Meier analysis with the log-rank test, the first 7-, 14-, and 28-day survival probabilities in sepsis patients with T2A ≤ 1 h were better than those for T2A > 1 h (Fig. 2). Similar results were observed for group stratification of T2A ≤ 1 h, between 1 and 2 h, between 2 and 3 h, and > 3 h, irrespective of 7-, 14-, and 28- day in-hospital mortality (Fig. 3).

### Non-linear Cox regression model

**Fig. 4:**
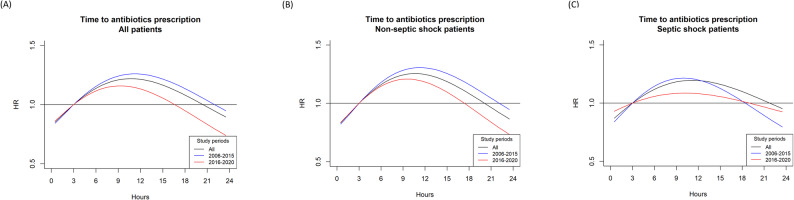
Nonlinear Cox regression curves illustrated the relationship between Time-to-Antibiotics (T2A) and the hazard ratio (HR) of in-hospital mortality. Black line: complete dataset; Blue line: 2006–2015 subset; Red line: 2016–2020 subset. (**A**) All sepsis patients. (**B**) Non-septic shock patients. (**C**) Septic shock patients. The curves demonstrate a U-shaped pattern, with the lowest HR at approximately 0.5 h and a progressive increase after ~ 3 h, consistent across subsets and clinical subgroups

Our results demonstrated that the risk (HR) of in-hospital mortality was decreased for T2A < 3 h and increased for T2A > 3 h, as presented in the heat map (Figure S2). Moreover, we did a sensitivity analysis by looking at various time restricted subsets and found the results to be similar across time intervals as demonstrated Figure S3. In all sepsis patients, with or without septic shock, the non-linear regression plot showed that the lowest HR was at 0.5 h (HR = 0.85, 95% CI 0.8–0.91) and the highest HR was at 10.5 h (HR = 1.22, 95% CI 1.12–1.32) (Fig. 4A).

We further dichotomized the sepsis patients into shock and non-shock groups. In the septic shock patients, the lowest HR was at 0.5 h (HR = 0.87, 95% CI 0.79–0.96) and the highest HR was at 11 h (HR = 1.21, 95% CI 1.06–1.35) (Fig. 4C). In the non-septic shock patients, the lowest HR was at 0.5 h (HR = 0.83, 95% CI 0.76–0.91) and the highest HR was at 10.5 h (HR = 1.25, 95% CI 1.12–1.41) (Fig. 4B). Figure 4 is plotted for time restricted subsets keeping in consideration the time span of the study to be long and again the findings were similar across time intervals.

## Discussion

The concept of time-to-antibiotics (T2A) refers to the interval within which empirical or targeted antimicrobial therapy should be administered to a patient with suspected or confirmed infection, irrespective of whether the precise source has been fully identified. In this study, using a comprehensive longitudinal database from a single tertiary medical center, we observed that patients who received antibiotics within 1 h (T2A ≤ 1 h) had a higher probability of in-hospital survival compared with those treated after 1 h. Traditional linear regression analyses were inconclusive. Consequently, we employed a non-linear Cox modeling approach to better characterize the time-dependent relationship between T2A and mortality. This analysis demonstrated a diminishing benefit as T2A approached 3 h, suggesting that antibiotic administration within this interval may represent a reasonable clinical safety margin for the general sepsis population. Beyond this threshold, the model showed a more pronounced increase in mortality risk, underscoring the importance of timely antibiotic administration. Notably, although propensity score methods are commonly recommended to address baseline imbalances, they were not be suitable for this research context. In emergency sepsis care, antibiotic administration is often deterministic in the sickest patients, violating the positivity assumption and making many high-acuity cases impossible to match. Additionally, unmeasured clinical judgment based on subtle physiological cues cannot be captured by propensity scores, potentially introducing bias rather than reducing it. For these reasons, we did not conduct propensity score matching and used covariate adjustment for our multivariable Cox regression which provided a more appropriate and clinically representative analytical framework for this study.

The findings from the nonlinear modelling offer a more nuanced understanding of how antibiotic timing influences mortality, beyond what linear models or categorical analyses can reveal. The nonlinear Cox regression demonstrated that the hazard of in-hospital mortality was lowest at approximately 0.5 h and remained relatively stable until around 3 h, after which the risk increased more noticeably. This consistent U-shaped pattern as demonstrated in Fig. 4, replicated across time-restricted subsets and in both septic shock and non-shock groups, suggests a clear phenomenon of diminishing returns, where early antibiotics confer the greatest benefit, but meaningful protection persists within the first three hours. Clinically, this indicates that while rapid antibiotic administration remains crucial, an approximate 3-hour safety margin is a realistic operational benchmark before mortality risk begins to rise substantially. These findings reinforce the “earlier is better” principle but also highlight the need to view antibiotic timing as one component of a broader resuscitation strategy rather than a singular determinant of outcome.

Several previous studies support our findings. Sterling et al. conducted a 2015 systematic review and meta-analysis including 16,178 sepsis patients, reporting that those receiving antibiotics more than 3 h after emergency triage (reference: T2A ≤ 3 h) had a pooled mortality OR of 1.16 [23, 24]. Among 11,017 patients with septic shock or severe sepsis, antibiotic administration more than 1 h after diagnosis (reference: T2A < 1 h) was associated with a pooled hospital mortality OR of 1.46. Similarly, a 2022 nationwide prospective study from Korea involving 3,035 patients found adjusted ORs of 0.78 for all sepsis patients and 0.66 for septic shock patients treated within 1 h [4]. The authors also noted a 35% increased mortality risk for each hour of delay within the first 3 h among septic shock patients, although this conclusion was derived from a non-linear model and should not be interpreted as reflecting a strictly linear effect. Seymour et al. further demonstrated in 2017 that more rapid completion of a 3-hour sepsis care bundle was associated with lower hospital mortality, with antibiotic timing exerting a greater impact than fluid resuscitation [25]. In our study, fluid administration variables were not included because more than 30% of patients developed acute respiratory failure during their ED stay, and 18.27%, who were excluded, had do-not-resuscitate orders. Moreover, fluid volume, vasopressor initiation, and timing of inotropic therapy were determined by individual clinicians, introducing substantial variability; therefore, these parameters were not incorporated into our analysis.

Due to the varying approaches in patient management across different hospitals, we expected that using a single, cohesive database from a single medical center would result in less data variability and bias. Therefore, a multi-center study design was not adopted in this study despite it being the popular academic trend in Taiwan. Through repeated internal validation of this hospital database, we are confident that our study can offer clinicians valuable and robust information. Our study period spans multiple eras of sepsis definitions, encompassing Sepsis-1 and Sepsis-2 through the introduction of Sepsis-3 in 2016. Because SIRS criteria served as the accepted clinical standard for diagnosing sepsis between 1998 and 2015, applying Sepsis-3 definitions retrospectively would have excluded numerous patients who were appropriately identified and treated according to contemporaneous practice, thereby introducing substantial retrospective definition bias. To preserve the epidemiologic integrity and historical validity of the cohort, we therefore applied the diagnostic criteria in use at the time of care. Recognizing, however, that the SOFA score provides stronger prognostic discrimination for mortality, we retrospectively calculated SOFA scores for all patients using raw physiological data and incorporated these as continuous covariates in our multivariable models to ensure rigorous adjustment for disease severity. This strategy balances fidelity to historical diagnostic standards with the advantages of modern risk adjustment. Although we anticipated concerns regarding our reliance on SIRS rather than SOFA for initial case identification, we believe that our principal findings would remain robust irrespective of the definition applied. As noted by Cortes-Puch et al., the SIRS criteria have been widely used in clinical practice and formed the foundation for numerous controlled trials, underscoring their continued relevance and methodological legitimacy [26].

Definitions of sepsis and septic shock vary considerably across studies investigating T2A. For example, Yunjoo et al. considered sepsis “recognized” when it appeared in the differential diagnosis list documented by the treating physician [4]. The authors also acknowledged that diagnosing septic shock within one hour using Surviving Sepsis Campaign (SSC) criteria was exceedingly difficult in routine practice. This is because septic shock patients typically require a sequence of time-dependent evaluations and interventions, including history taking, laboratory testing, blood culture acquisition, initiation of life-support measures, fluid resuscitation, and potential vasopressor or inotropic therapy, before antibiotic administration can occur. These necessary clinical processes inevitably delay the ability to deliver antibiotics within one hour in many septic shock cases, contributing to heterogeneity in T2A definitions and operational feasibility across studies.

In this study, T2A was measured from ED arrival or triage as the starting point for sepsis care, according to the ACEP guideline; however, the SSC guideline (2021 versions) uses the starting time of bundle care of T2A as the time of sepsis “recognition” [27]. The Infectious Diseases Society of America does not have a firm recommendation for defining the zero-time point for T2A. It was suggested that the zero point should depend on the clinical setting. Hence, prospective studies on the feasibility, meaningfulness, and reproducibility of different definitions of time zero are warranted. In the current study, we adopted the ACEP guideline for defining the starting point. While the SSC guideline definition of T2A may have been more relevant, it is much more difficult to track in clinical practice, since most cases of sepsis develop insidiously [11, 27]. A potential source of treatment-selection bias should be acknowledged where, patients receiving antibiotics within 1 h tend to present with higher lactate levels, worse renal function, and more frequent septic shock, suggesting clinicians may have fast-tracked the most critically ill patients. As such bias would typically attenuate rather than exaggerate the observed benefit of early antibiotics, we adjusted for illness severity using SOFA scores and other covariates. The persistence of a significant association after adjustment indicated that the observed relationship is unlikely to be explained solely by preferential treatment of sicker patients.

A strength of this study was the utilization of a large longitudinal cohort which went through every SSC guideline revision. The database for this cohort represents a large amount of clinical data from the hospital electronic health record system, including detailed information on sepsis courses since “Early Goal-Directed Therapy” was proposed in 2001 by Emanuel Rivers et al. [28]. Since then, quality incentive programs regarding sepsis have been implemented annually by Taiwan’s National Health Insurance Bureau, with generous rewards given to hospitals that achieve the benchmark values. Data were analyzed by both linear and non-linear regression models, as this has been shown to be necessary for describing the complex patterns of data that emerge from the outcomes of sepsis treatment.

This study has several limitations. First, while the single-center design of this study reduces variability, it may limit the applicability of these findings to other healthcare systems with different sepsis management protocols; however, it also provides a cohesive dataset reflecting a consistent treatment strategy. Second, Time Zero was defined using the ED triage timestamp rather than the moment of clinical recognition, which is difficult to determine reliably in retrospective data. Although this approach yields a reproducible “door-to-antibiotic” interval aligned with Taiwan’s time-sensitive emergency workflows, the true recognition-to-treatment interval is likely shorter allowing this approach to enhance reproducibility. Third, diagnostic criteria for sepsis evolved over the study period, and reliance on SIRS, known for high sensitivity but lower specificity—introduces potential misclassification bias; however, SIRS was the accepted clinical standard during most of the study period, and applying Sepsis-3 criteria retrospectively would have excluded many appropriately treated patients, thereby introducing retrospective definition bias. We mitigated this by retrospectively calculating SOFA scores and incorporating them as continuous covariates to adjust for severity. Fourth, patients were excluded for inappropriate antibiotic therapy (IAAT) based on clinical assessment, but because IAAT exclusions accounted for only ~ 1% of the cohort, their impact on overall findings is minimal. Fifth, we lacked treatment-response metrics such as lactate clearance or capillary refill time normalization, making it difficult to determine whether earlier antibiotics directly reduced mortality or whether T2A partly reflects earlier recognition and more comprehensive resuscitation, however, the consistent association across multiple analyses suggests that timing likely remains an important contributing factor, despite the potential for residual confounding. Finally, the inability to distinguish viral from bacterial sepsis across the 24-year study period, as viral diagnostics were inconsistently available and often performed selectively. Any etiologic classification would therefore carry substantial misclassification risk, and our analyses focused on suspected sepsis at presentation, consistent with ED practice where empiric antibiotics are initiated before pathogen confirmation. However, variations in underlying etiology, especially viral sepsis, may account for some differences in mortality, and acknowledging this helps clarify the scope of our findings and highlights an important direction for future research.

## Conclusion

In this large retrospective cohort, shorter T2A was associated with lower in-hospital mortality among patients with sepsis and septic shock, with the most favorable estimates observed within the first hour and a diminishing association up to approximately 3 h. While this pattern was consistently observed in our study, it represents an associative finding and warrants further validation in prospective studies before being incorporated into clinical practice recommendations.

## Supplementary Information

Below is the link to the electronic supplementary material.

Supplementary Material 1

## Data Availability

The data that support the findings of this study are available from the involved hospitals, mentioned in the manuscript, but restrictions apply to the availability of these data, which were used under license for the current study, and so are not publicly available. Data are however available from the authors upon reasonable request and with permission of the involved hospitals.

## Abbreviations

SSC: Surviving Sepsis Campaign
T2A: Time to antibiotics
ED: Emergency Department
ACEP: American College of Emergency Physicians
SOFA: Sequential Organ Failure Assessment
IAAT: Inappropriate antibiotics treatment
MDR: Multi-drug resistant
CCI: Charlson comorbidity index
ICU: Intensive care unit
WBC: White blood cell
CRP: C-reactive protein
SD: Standard deviation
CI: Confidence Interval
AIC: Akaike Information Criterion
HR: Hazard ratio
OR: Odds ratio
