# Supplementary material for: Optimal timing of antibiotics administration for sepsis or septic shock in the emergency department
Source: BMC Emerg Med. 2026 Jan 19;26:47. doi: 10.1186/s12873-026-01471-5 (PMC12884633; doi:10.1186/s12873-026-01471-5)
Supplement: Supplementary file 1 — Supplementary Material 1 [file 12873_2026_1471_MOESM1_ESM.docx]

**Supplementary Materials**

**Optimal Timing of Antibiotics Administration for Sepsis or Septic Shock in the Emergency Department**

**Supplementary Figures**


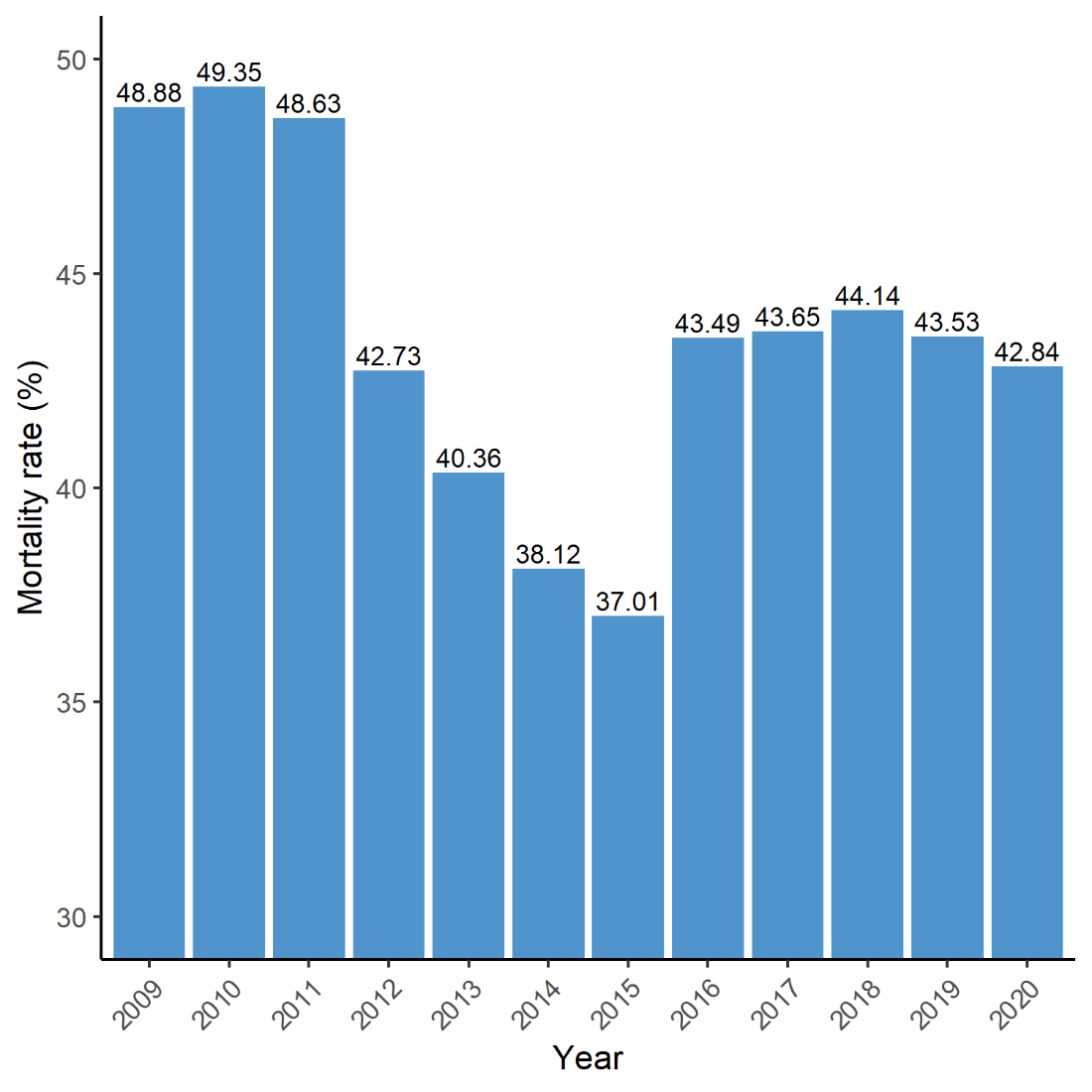


**Figure S1.** **Annual in-hospital mortality rate in sepsis patients, 2009-2020.**


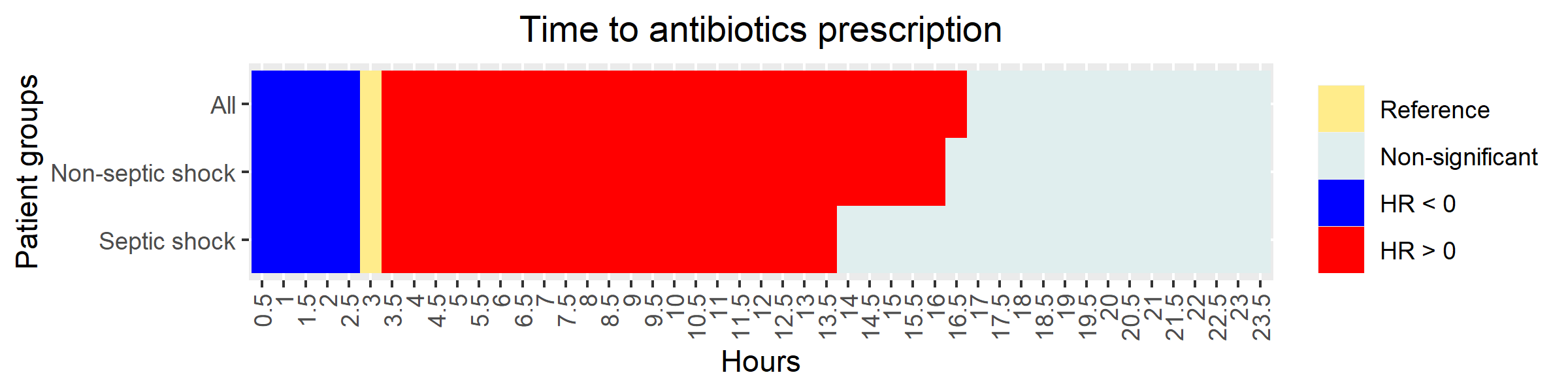


**Figure S2. Heat map describing the optimal timing of antibiotics administration in the non-linear model, that is, within 3 hours, no matter whether in septic shock or non-shock patients.**


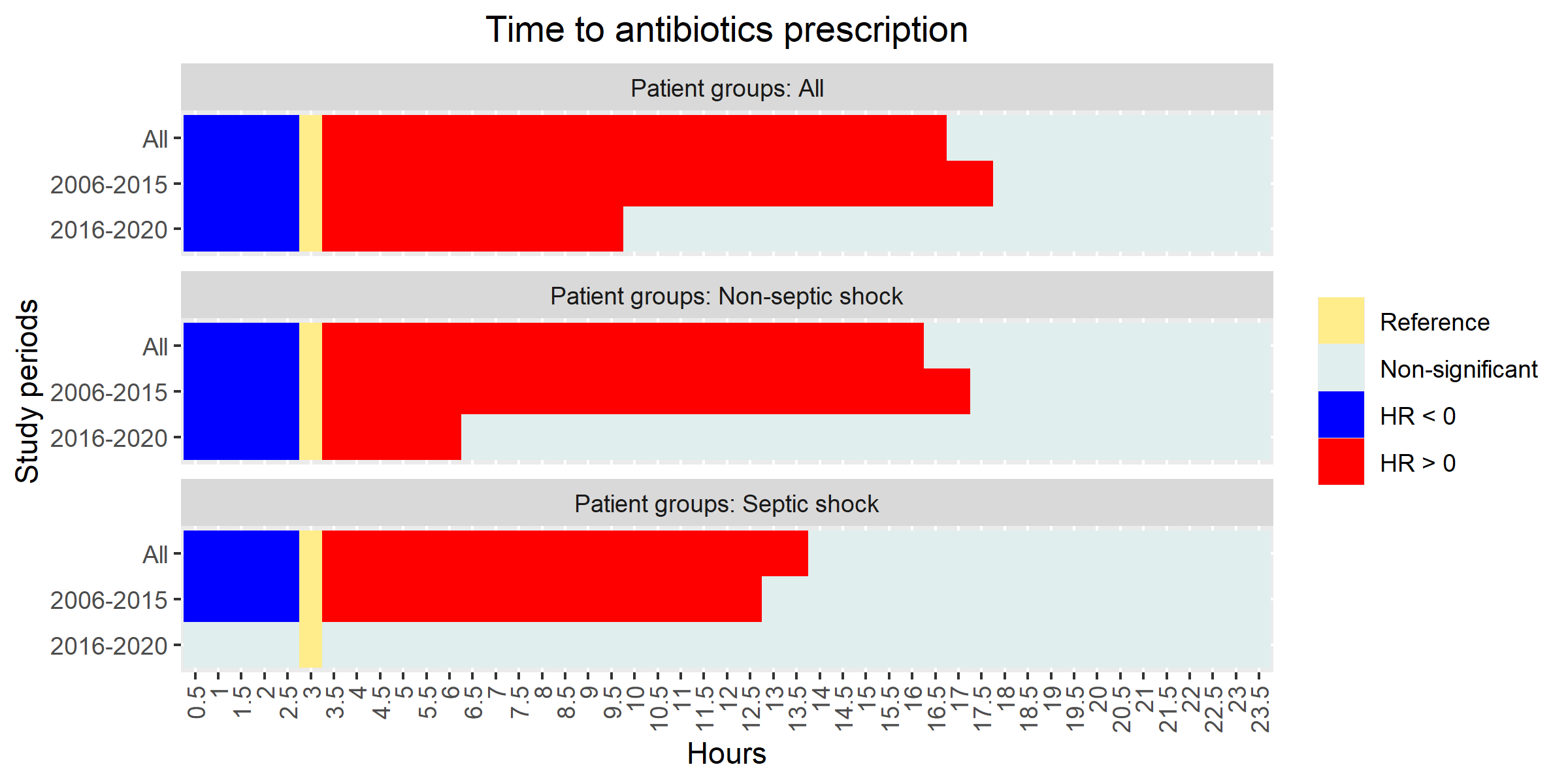


**Figure S3. Heat map describing the optimal timing of antibiotics administration in the non-linear model for time restricted subsets, that is, within 3 hours, irrespective of whether in septic shock or non-shock patients.**
